# Excessive glucocorticoid-induced muscle MuRF1 overexpression is independent of Akt/FoXO1 pathway

**DOI:** 10.1042/BSR20171056

**Published:** 2017-11-17

**Authors:** Xiao Juan Wang, Jing Jing Xiao, Lei Liu, Hong Chao Jiao, Hai Lin

**Affiliations:** Shandong Provincial Key Laboratory of Animal Biotechnology and Disease Control and Prevention, Department of Animal Science, Shandong Agricultural University, 61 Daizong Street, Taian City, Shandong Province 271018, China

**Keywords:** FoXO1, glucocorticoid, MuRF1, muscle cell, protein metabolism

## Abstract

The ubiquitin-proteasome system (UPS)-dependent proteolysis plays a major role in the muscle catabolic action of glucocorticoids (GCs). Atrogin-1 and muscle-specific RING finger protein 1 (MuRF1), two E3 ubiquitin ligases, are uniquely expressed in muscle. It has been previously demonstrated that GC treatment induced MuRF1 and atrogin-1 overexpression. However, it is yet unclear whether the higher pharmacological dose of GCs induced muscle protein catabolism through MuRF1 and atrogin-1. In the present study, the role of atrogin-1 and MuRF1 in C2C12 cells protein metabolism during excessive dexamethasone (DEX) was studied. The involvement of Akt/forkhead box O1 (FoXO1) signaling pathway and the cross-talk between anabolic regulator mammalian target of rapamycin (mTOR) and catabolic regulator FoXO1 were investigated. High concentration of DEX increased MuRF1 protein level in a time-dependent fashion (*P*<0.05), while had no detectable effect on atrogin-1 protein (*P*>0.05). FoXO1/3a (Thr24/32) phosphorylation was enhanced (*P*<0.05), mTOR phosphorylation was suppressed (*P*<0.05), while Akt protein expression was not affected (*P*>0.05) by DEX. RU486 treatment inhibited the DEX-induced increase of FoXO1/3a phosphorylation (*P*<0.05) and MuRF1 protein; LY294002 (LY) did not restore the stimulative effect of DEX on the FoXO1/3a phosphorylation (*P*>0.05), but inhibited the activation of MuRF1 protein induced by DEX (*P*<0.05); rapamycin (RAPA) inhibited the stimulative effect of DEX on the FoXO1/3a phosphorylation and MuRF1 protein (*P*<0.05).

## Introduction

Skeletal muscle is an essential component of the body. The maintenance of skeletal muscle mass is of paramount importance for systemic energy homeostasis [1]. The muscle mass usually results from a dynamic balance between protein synthesis and catabolism [2]. When the body is suffering from severe stress, excessive glucocorticoids (GCs) in circulation cause muscular loss by impeding protein synthesis and enhancing protein breakdown [3–5]. GCs, as the final effectors of the hypothalamic–pituitary–adrenal axis, participate in the maintenance of whole-body homeostasis. Many pathological conditions, including muscle atrophy, result from an increase in circulating GC levels [6].

The increased muscular loss during stress is mainly caused by activation of proteolytic systems [7]. Amongst these proteolytic systems, ubiquitin-proteasome system (UPS) is considered to play a major role in the catabolic action of GCs [8]. The stimulation of UPS is mediated through the increased expression of several atrogenes, such as atrogin-1 and muscle-specific RING finger protein 1 (MuRF1), which are two E3 ubiquitin ligases involved in the targetting of protein to be degraded by the proteasome machinery [8]. MuRF1 and atrogin-1 are uniquely expressed in muscle [9,10]. Muscle wasting is associated with an increase in circulating GC levels [11]. Dexamethasone (DEX), a synthetic GC that is specific for the GC receptor (GR) and delayed plasma clearance [12], is widely used in studies of muscle atrophy in C2C12 cells. In the previous study, it has been demonstrated that DEX treatment (0.05–10 µmol/l) induced MuRF1 and atrogin-1 overexpression [13–15]. However, it is yet unclear whether the higher pharmacological dose of DEX induced catabolic effect on skeletal muscle through MuRF1 and atrogin-1.

The forkhead transcriptional factor subfamily forkhead box O1 (FoxO1) plays essential roles in the transcriptional cascades that are responsible for metabolism in the muscle, liver, brain, pancreas, and adipose tissues [16–19]. Both the translocation and activity of FoXO1 are required for up-regulation of atrogin-1 and MuRF1. FoxO1 is an important downstream target of the PI3K/Akt pathways [20]. Activated Akt phosphorylates FoXO1, leading to its nuclear exclusion and proteasomal degradation [21,22]. We thus hypothesize that Akt/FoXO1 signaling pathway is required in the ubiquitin ligase expression and in the dysregulation of muscular protein metabolism induced by excessive GCs.

In this study, we investigated whether excessive GCs enhanced proteolysis through the MuRF1 and atrogin-1, and the involvement of Akt/FoXO1 signaling pathway was also evaluated. According to previous report [23] and our recent studies [24,25], a hyperglucocorticoid milieu (100 µmol/l, DEX) in C2C12 cells suppressed protein synthesis and enhanced proteolysis. In the present study, 100 µmol/l DEX was employed to induce a hyperglucocorticoid milieu.

## Materials and methods

### Cell culture and *in vitro* treatments

Murine C2C12 cells (CCTCC, Wuhan, China) were cultured in DMEM (HyClone, Logan, UT, U.S.A.) supplemented with 10% FBS (HyClone, Logan, UT, U.S.A.), 100 U/ml penicillin, and 100 µg/ml streptomycin (Solarbio, Shanghai, China) and maintained at 37°C in a humidified atmosphere containing 5% CO_2_. When cells were 70% confluent, the proliferation medium was replaced with DMEM containing 2% horse serum (Gibco, U.S.A.) for inducing myogenic differentiation. After 84 h, the cultures were shifted to serum-free DMEM for 12 h [25].

#### Trial 1: DEX and its time-dependent effect

After a 12-h incubation in serum-free medium, cells were exposed to DMEM-LM (Thermo, Fremont, CA, U.S.A.) with or without 100 µmol/l DEX [23–25] and collected after 3, 8, 12, and 24 h, for the analysis of mRNA expression by RT-PCR and protein expression by immunoblotting.

#### Trial 2: DEX and epoxomicin (proteasome inhibitor) treatment

After a 12-h incubation in serum-free medium, cells were exposed to one of the four following treatments: basal serum-free medium (Control); basal medium with 100 µmol/l DEX for 8 h (DEX); basal medium with 1.5 µmol/l epoxomicin (EPOX) for 5 h; basal medium with 1.5 µmol/l EPOX for 5 h prior to addition of 100 µmol/l DEX for 8 h (DEX + EPOX). Following this, all cells were collected and subjected to the further analysis.

#### Trial 3: DEX and RU486 (GR inhibitor) treatment

After a 12-h incubation in serum-free medium, cells were exposed to one of the four following treatments: basal serum-free medium (Control); basal medium with 100 µmol/l DEX for 8 h (DEX); basal medium with 10 µmol/l RU486 for 5 h (RU486); basal medium with 10 µmol/l RU486 for 5 h prior to addition of 100 µmol/l DEX for 8 h (DEX+RU486). Following this, all cells were collected and subjected to the further analysis.

#### Trial 4: DEX and LY294002 (PI3K inhibitor) treatment

After a 12-h incubation in serum-free medium, cells were exposed to one of the four following treatments: basal serum-free medium (Control); basal medium with 100 µmol/l DEX for 8 h (DEX); basal medium with 10 µmol/l LY294002 for 5 h (LY); basal medium with 10 µmol/l LY294002 for 5 h prior to addition of 100 µmol/l DEX for 8 h (DEX+LY). Following this, all cells were collected and subjected to the further analysis.

#### Trial 5: DEX and rapamycin (mammalian target of rapamycin inhibitor) treatment

After a 12-h incubation in serum-free medium, cells were exposed to one of the four following treatments: basal serum-free medium (Control); basal medium with 100 µmol/l DEX for 8 h (DEX); basal medium with 25 µmol/l rapamycin (RAPA) for 5 h; basal medium with 25 µmol/l RAPA for 5 h prior to addition of 100 µmol/l DEX for 8 h (DEX+RAPA). Following this, all cells were collected and subjected to the further analysis.

### Protein preparation and Western blot analysis

Cell samples were homogenized on ice in radioimmunoprecipitation lysis buffer (50 mmol/l Tris/HCl at pH 7.4, 1% NP-40, 0.25% sodium deoxycholate, 150 mmol/l NaCl, 1 mmol/l EDTA, 1 mmol/l phenylmethylsulfonyl fluoride, 1 µg/ml aprotinin, 1 µg/ml leupeptin, 1 µg/ml pepstatin, 1 mmol/l sodium orthovanadate, 1 mmol/l sodium fluoride) and centrifuged at 12000 ***g*** for 5 min at 4°C. Protein concentration was determined using the BCA assay kit (Beyotime, Jiangsu, China) according to the manufacturer’s protocol. After boiling at 100°C for 5 min, the protein extracts were electrophoresed in 7.5–10% SDS polyacrylamide gels (Bio-Rad, Richmond, CA) according to the procedure described by Laemmli [26]. The separated proteins were then transferred on to a nitrocellulose membrane in Tris-glycine buffer containing 20% methanol at 4°C. The membranes were blocked and then probed with primary antibodies including anti-MuRF1, anti-atrogin-1, anti-phospho-FoxO1/3a (Thr24/32) (Abcam, Cambridge, U.K.), anti-FoxO1, anti-p-mTOR (mammalian target of RAPA) (Ser^2448^), anti-mTOR (Cell Signaling Technology, Beverly, U.S.A.), anti-p-Akt (Ser^473^), and anti-Akt (Beyotime, Jiangsu, China). Protein detection was performed using goat anti-rabbit IgG (H + L)-HRP conjugated secondary antibody (Bio–Rad, Richmond, CA) or HRP-labeled goat anti-mouse IgG (H + L) secondary antibody (Beyotime, Jiangsu, China) with ECL using Western blot detection reagents (Beyotime, Jiangsu, China). β-actin was used as a loading control (Beyotime, Jiangsu, China). Western blots were developed and quantified using BioSpectrum 810 with VisionWorksLS 7.1 software (UVP LLC, Upland, CA).

### RNA preparation and analysis

Total RNA extraction and real-time PCR were performed as described previously [25]. Primers were designed to span an intron to avoid genomic DNA contamination. Standard curves were generated using pooled cDNA from the samples that were assayed and to calculate the efficiency of the real-time PCR primers. The primers were as follows: Atrogin-1: forward, GCTGGATTGGAAGAAGAT and reverse, GAGAATGTGGCAGTGTTTG; MuRF1 forward, CTGGAGGTCGTTTCCGTTGC and reverse, TCGGGTGGCTGCCTTTCTGC; FoXO1: forward, GAGTGGATGGTGAAGAGCGT and reverse, GGGACAGATTGTGGCGAAT; FoXO3: forward, CCCTAACCCAGCAGAGACTGT and reverse, GGAAACAAACACAAGACGACACT; β-actin: forward, ACCACACCTTCTACAATGAG and reverse, ACGACCAGAGGCATACAG. Primer against β-actin was used as internal controls. The comparative CT method (**2^−ΔΔCT^**) was used to quantitate mRNA expression, according to Livak and Schmittgen method [27]. All samples were included in the same assay for one gene to avoid interassay variability.

### Statistical methods

The data are presented as the mean ± S.E.M. All the data were subjected to one-way ANOVA analysis performed with the Statistical Analysis Systems statistical software package (Version 8e, SAS Institute, Cary, NC, U.S.A.) to test the main effect of treatments. The homogeneity of variances amongst treatments was confirmed using Bartlett’s test (SAS Institute). When the primary effect of treatment was significant, differences between means were assessed by Duncan’s multiple range analysis. Means were considered significantly different at *P*<0.05.

## Results

### The time-dependent effect of DEX treatment

**Figure 1:**
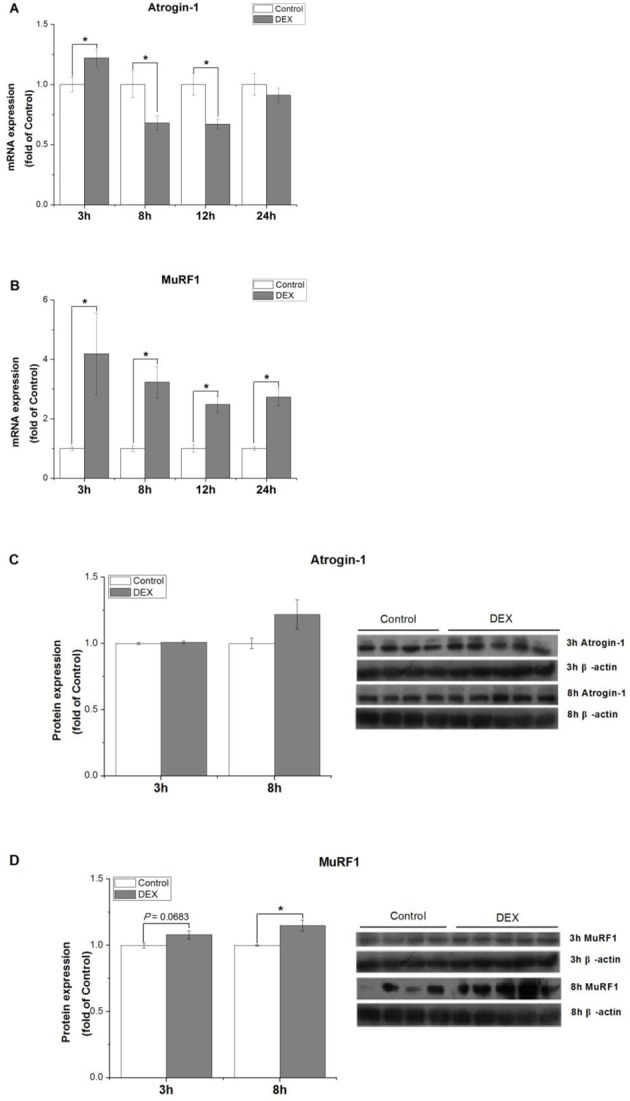
The time-dependent effect of DEX treatment The effect of DEX treatment (100 µM for 3, 8, 12, and 24 h) on atrogin-1 (**A, C**) and MuRF1 (**B, D**) expressions in C2C12 cells. Results are presented as the mean ± S.E.M. (*n*≥4); control and DEX treatments were compared at each time point, and means with * are significantly different (*P*<0.05).

**Figure 2:**
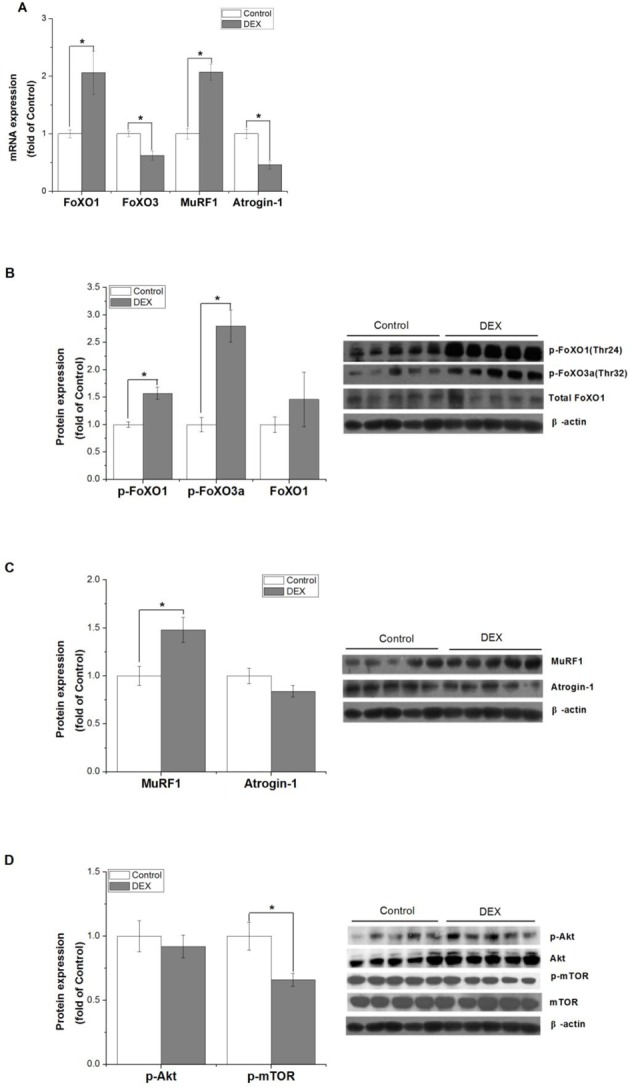
Dex effects on protein degradation regulators and protein synthesis regulators The effect of DEX treatment (100 µM for 8 h) on mRNA expressions of FoXO1, FoXO3, MuRF1, and atrogin-1 (**A**), protein expressions of p-FoXO1/3a (**B**), MuRF1, atrogin-1 (**C**), p-mTOR and p-Akt (**D**) in C2C12 cells. Results are presented as the mean ± S.E.M. (*n*≥5); control and DEX treatments were compared for each gene, and means with * are significantly different (*P*<0.05).

The effect of high concentration of DEX on the UPS was investigated. Atrogin-1 mRNA level was significantly increased at 3 h (*P*<0.05, Figure 1A), decreased at 8 h (*P*<0.05, Figure 1A, 2A) and 12 h (*P*<0.05, Figure 1A), and not changed at 24 h of DEX (*P*>0.05, Figure 1A), whereas atrogin-1 protein level was not significantly changed by DEX at 3 h (*P*>0.05, Figure 1C) and 8 h (*P*>0.05, Figure 1C and 2C). DEX treatment significantly up-regulated the mRNA level of MuRF1 at 3 h (*P*<0.05, Figure 1B), 8 h (*P*<0.05, Figure 1B and 2A), 12 h (*P*<0.05, Figure 1B), and 24 h (*P*<0.05, Figure 1B), and up-regulated the protein level of MuRF1 at 8 h (*P*<0.05, Figure 1D and 2C) but not at 3 h (*P*>0.05, Figure 1D).

The ubiquitin ligase MuRF1 was dramatically regulated by DEX treatment at 8 h; therefore, we then detected the DEX effect at 8 h. The upstream pathway of atrogin-1 and MuRF1, Akt/FoXO1, was then measured. DEX treatment increased the FoXO1 mRNA and decreased FoXO3 mRNA (*P*<0.05, Figure 2A), while increased FoXO1/3a (Thr24/32) phosphorylation (*P*<0.05, Figure 2B). Akt protein was not affected by DEX (*P*>0.05, Figure 2D). As a crucial protein synthesis regulator, mTOR phosphorylation was suppressed by DEX (*P*<0.05, Figure 2D).

### Effect of proteasome inhibition with EPOX on DEX-induced effects

**Figure 3:**
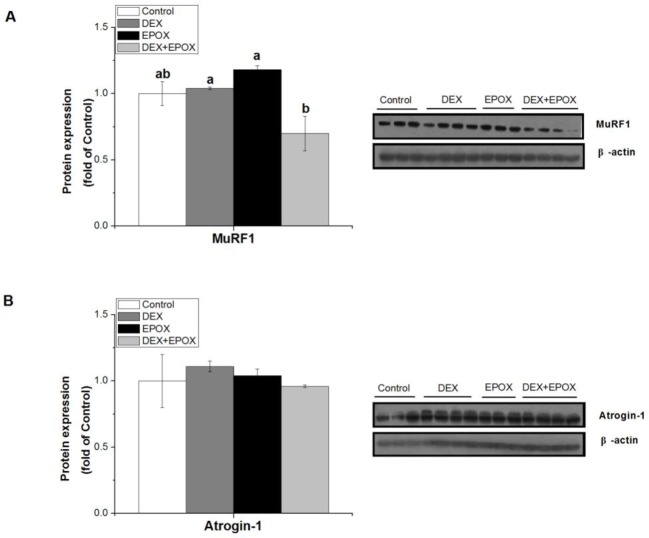
Effect of proteasome inhibition with EPOX on DEX-induced effects The effect of DEX (100 µM for 8 h) and EPOX (1.5 μM for 5 h) on protein expressions of MuRF1 (**A**) and atrogin-1 (**B**) in C2C12 cells. Results are presented as the mean ± S.E.M. (*n*≥3); four groups were compared for each gene, and means without a common letter are significantly different (*P*<0.05).

To further confirm the role of ubiquitin ligases in UPS pathway during GC treatment, EPOX was used to inhibit proteasome in C2C12 cells. MuRF1 protein expression was suppressed by DEX + EPOX compared with DEX treatment alone (*P*<0.05, Figure 3A). However, DEX and EPOX had no influence on the atrogin-1 protein expression (*P*>0.05, Figure 3B).

### Effect of GR blockage with RU486 on DEX-induced effects

**Figure 4:**
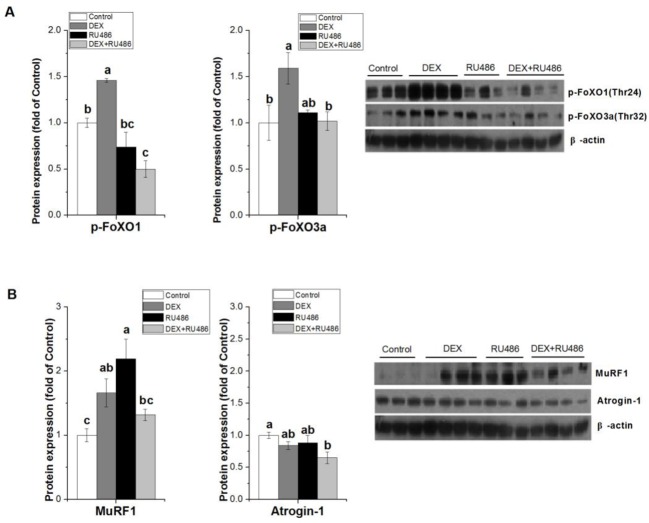
Effect of GR blockage with RU486 on DEX-induced effects The effect of DEX (100 µM for 8 h) and RU486 (10 μM for 5 h) on protein expressions of p-FoXO1/3a (**A**), MuRF1 and atrogin-1 (**B**) in C2C12 cells. Results are presented as the mean ± S.E.M. (*n*≥3); four groups were compared for each gene, and means without a common letter are significantly different (*P*<0.05).

To further explore the mechanism underlying GC effect on UPS pathway, RU486 was used to inhibit GR in C2C12 cells. DEX-induced increase of FoXO1/3a phosphorylation was significantly inhibited by DEX+RU486 treatment (*P*<0.05, Figure 4A), and DEX-induced increase of MuRF1 protein was partially attenuated (−20.5%) by DEX+RU486 (Figure 4B). However, DEX and RU486 had no influence on the atrogin-1 protein expression (*P*>0.05, Figure 4B).

### Effect of PI3K blockage with LY294002 on DEX-induced effects

**Figure 5:**
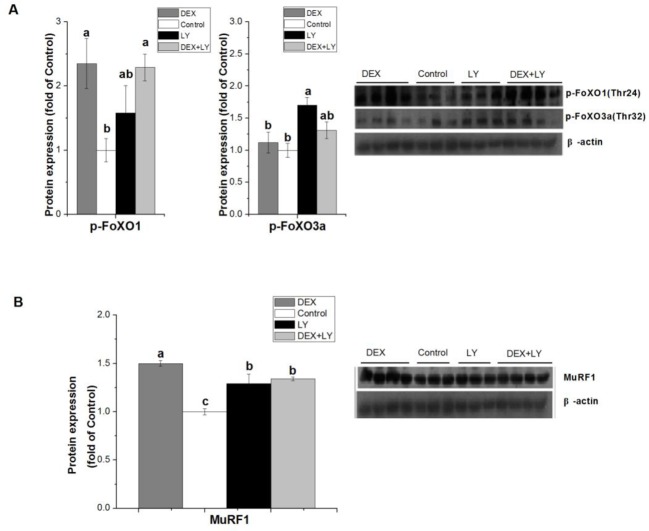
Effect of PI3K blockage with LY294002 on DEX-induced effects The effect of DEX (100 µM for 8 h) and LY294002 (10 μM for 5 h) on protein expressions of p-FoXO1/3a (**A**) and MuRF1 (**B**) in C2C12 cells. Results are presented as the mean ± S.E.M. (*n*≥3); four groups were compared for each gene, and means without a common letter are significantly different (*P*<0.05).

To further explore the effect of PI3K/Akt on UPS pathway, LY294002 was used to inhibit PI3K in C2C12 cells. DEX+LY294002 did not restore the stimulative effect of DEX on the FoXO1/3a phosphorylation (*P*>0.05, Figure 5A), but inhibited the activation of MuRF1 protein induced by DEX (*P*<0.05, Figure 5B).

### Effect of mTOR blockage with RAPA on DEX-induced effects

**Figure 6:**
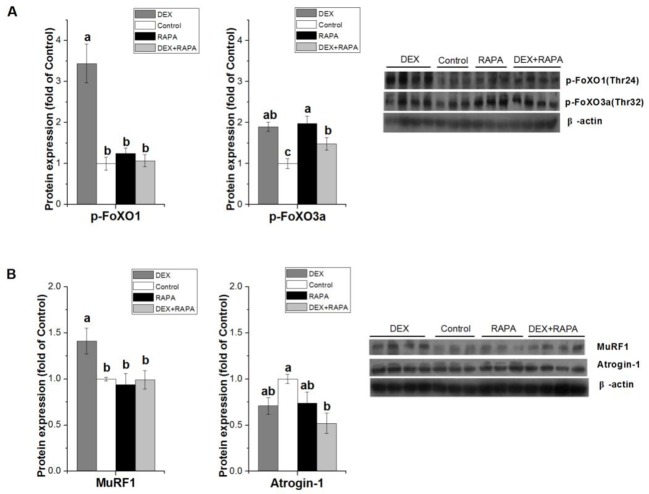
Effect of mTOR blockage with RAPA on DEX-induced effects The effect of DEX (100 µM for 8 h) and RAPA (25 μM for 5 h) on protein expressions of p-FoXO1/3a (**A**), MuRF1 and atrogin-1 (**B**) in C2C12 cells. Results are presented as the mean ± S.E.M. (*n*≥3); four groups were compared for each gene, and means without a common letter are significantly different (*P*<0.05).

To further explore the effect of mTOR on UPS pathway, RAPA was used to inhibit mTOR in C2C12 cells. DEX + RAPA inhibited the stimulative effect of DEX on the FoXO1 phosphorylation and MuRF1 protein (*P*<0.05, Figure 6A,B). However, DEX and RAPA had no detectable effect on atrogin-1 protein level (*P*>0.05, Figure 6B).

## Discussion

**Figure 7:**
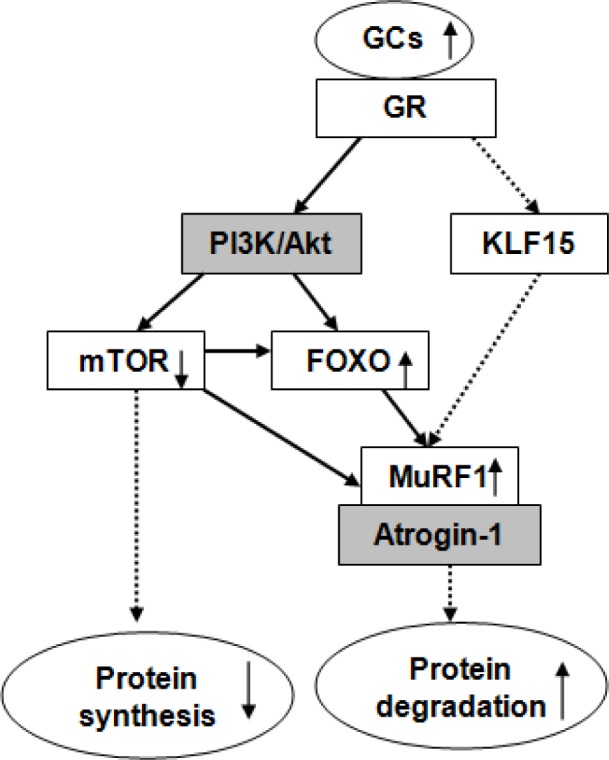
Proposed model of excessive GC action on protein synthesis and protein degradation in C2C12 cells (↑ increase; ↓ decrease; gray blocks represent unaffected proteins; solid arrows represent findings in this study and dashed arrows represent findings reported previously [23–25,38,39]). Excessive GCs stimulated protein degradation through MuRF1, but not atrogin-1. Other pathway that is independent of Akt/FoXO1 pathway may account for the excessive GC-induced MuRF1 activation. There is a cross-talk between anabolic regulator mTOR and catabolic regulator FoXO1-MuRF1.

The previous report [23] and our recent studies [24,25] have demonstrated that excessive GCs (100 µmol/l DEX) suppressed muscle protein synthesis and enhanced proteolysis. On the basis of above studies, the role of ubiquitin ligases in protein metabolism during excessive GC exposure was investigated in the present study. In line with our previous study, excessive GCs stimulated UPS through MuRF1, but not atrogin-1. We novelly find that other pathway that is independent of Akt/FoXO1 pathway may account for the excessive GC-induced MuRF1 activation. The cross-talk between anabolic regulator and catabolic regulator is also noteworthy. Proposed model of excessive GC action on protein metabolism in C2C12 cells is shown in Figure 7.

### The stimulation by excessive GCs of the UPS is mediated through MuRF1, but not atrogin-1

The catabolic effect and muscle atrophy caused by GCs have been well studied in mammals [28] and in chickens [29]. Muscle mass loss occurs when the degradation rate is higher than the synthesis rate. Amongst three proteolytic systems (lysosomal, proteasomal, and calpain dependent), UPS is considered to play a major role in the catabolic action of GCs [8]. We thus assessed two ubiquitin ligases, atrogin-1 and MuRF1, in the present study. At the early stage of DEX exposure (3 h), the increased mRNA levels of atrogin-1 and MuRF1 in the present study were in agreement with previous study that also used 100 µmol/l DEX for 4 h [30]. At 8–24 h, we found that MuRF1, but not atrogin-1, was increased by DEX at both transcriptional and translational levels; the results are in line with our recent study [24], but contrary to many other studies where atrogin-1 was up-regulated by DEX with lower dose [13–15,30], indicating a dose- and time-dependent manner of GC effect. The role of MuRF1 is further confirmed, as we find MuRF1 expression was suppressed by DEX+proteasome inhibitor compared with DEX. However, DEX and proteasome inhibitor had no influence on the atrogin-1.

Adrenal GCs produce their actions via a signal pathway involving the ubiquitously expressed GR. Upon binding GCs, GR translocates into the nucleus and binds to the GC response element (GRE) in the promoters of target genes. It has been reported that the promoter regions of MuRF1 [31], but not atrogin-1 [32], contain functional GREs. Consistently, in the present study, MuRF1 expression was significantly elevated during DEX treatment, and was partially attenuated after GR inhibition, while atrogin-1 was not significantly increased by DEX. The results suggest the stimulation by GCs of the UPS is mediated mainly through MuRF1, but not atrogin-1. In other studies using DEX with lower dose [13–15,30], however, atrogin-1 expression was also increased by DEX. We thus speculate the lower concentrations of GCs up-regulate MuRF1 and atrogin-1 through GR and non-GR-dependent mechanism respectively, whereas the higher concentrations of GCs only up-regulate MuRF1 through GR-dependent mechanism.

### Other pathway that is independent of Akt/FoXO1 pathway may account for the excessive GC-induced MuRF1 overexpression

Several studies have demonstrated that muscle-specific ubiquitin ligase MuRF1 is the target of FoxO1, and FoxO1 inhibition prevents MuRF1 activation [32,33]. The proximal promoter of the FoxO1 gene contains multiple functional GREs and FoxO1 gene expression is regulated by binding of the GREs [34]. The reduced phosphorylation is an important mechanism of FoxO1 activation [35]. We thus investigated whether FoxO1/3a phosphorylation is associated with MuRF1 expression during high dose of DEX treatment. In line with previous study, FoxO1 mRNA was up-regulated by DEX [34,36]. The phosphorylation of FoXO1/3a was increased by DEX, suggesting that GCs induced the inactivation of FoxO1. Furthermore, the increases of FoXO1/3a phosphorylation by DEX were inhibited after RU486 treatment, indicating that GC effect is a GR-driven mechanism. The suppression of FoxO1 pathway by DEX was in line with the previous studies [24,28]. However, the up-regulated MuRF1 mRNA and protein by DEX indicated the activated muscle atrophy-related genes, consistent with previous results [28,34,36]. These results implied that FoxO1 is not a major factor contributing to the GC-induced MuRF1 overexpression.

FoxO1 is an important downstream target of the PI3K/Akt pathways [20,37]. FoxO1 phosphorylation by Akt leads to exclusion of FoxO1 from the nucleus and the phosphophorylated FoxO1 is ubiquitinated in the cytoplasm followed by degradation [22]. In the present results, unexpectedly, Akt expression was not affected by DEX; protein levels of phospho-FoXO1/3a were not affected when adding PI3K/Akt inhibitor compared with DEX treatment alone, suggesting PI3K/Akt is not necessary in GC-induced FoXO1 phosphorylation. Although the involvement of Akt/FoxO1 transcription factors is well known in the gene regulation of MuRF1 under the presence of excessive GCs [32,33], a recently discovered transcription factor that promotes skeletal muscle atrophy via transcriptional regulation of atrogenes in GC treatment has been reported. According to the study of Shimizu et al. [38], the up-regulation of MuRF1 in response to DEX might be mediated through an Akt/FoXO-independent pathway, such as Kruppe-like factor 15 (KLF15) transcription factor – overexpression of KLF15 causes myotube atrophy in an Akt-independent manner [39]; KLF15 co-operating with FoxO significantly enhanced the promoter activity of atrogenes [38]. All these results implicate that Akt and FoXO1 were not essential in the regulation of MuRF1 by GCs. It is likely that an Akt/FoXO1-independent pathway, such as KLF15, up-regulates the expression of ubiquitin ligases MuRF1 after DEX treatment. The present result is noteworthy, and is in fact contrary to the known effects of DEX on Akt and FoXO1 expression [15]. One possible explanation is the difference in DEX dose.

### Cross-talk between mTOR and FoXO1-MuRF1 pathways

Excessive GCs cause muscular loss by impeding protein synthesis and enhancing protein breakdown [5]. The mTOR pathway plays a major role in the regulation of skeletal muscle fiber size [40–42]. In the present study, mTOR phosphorylation was depressed by DEX, consistent with our recent study in C2C12 cell, which indicated that DEX repressed protein synthesis rate through the involvement of mTOR pathways [24,25]. Moreover, DEX enhanced myostatin-dependent muscle proteolysis in our recent study [24], and increased the expression of atrophy-related gene MuRF1 in the present study as well as our recent report [24]. All these results imply that GCs facilitate proteolysis meanwhile inhibiting protein synthesis and further exacerbating the loss of protein and muscle mass.

Given GCs facilitated muscle proteolysis meanwhile blunting muscle protein synthesis, we speculate there are associations between atrophy-related genes and anabolic regulators. We thus investigated whether MuRF1 is affected by mTOR signaling, using mTOR inhibitor RAPA. Much of the evidence suggests that there is a cross-talk between mTOR and FoXO1-MuRF1 pathways. mTOR signaling could be inhibited directly by FoXO1 [43], whereas there is a disagreement on the involvement of mTOR in proteolysis and muscle atrophy. mTOR is considered to be a master regulator of autophagy, and the mTOR inhibitor RAPA induces autophagy in many cell systems [44]; the mTOR pathway is necessary for mediating mRNA changes of atrogenes during hypertrophy [45]; mTORC2 is required for signaling to Akt-FoXO [46]; inhibition of Rictor causes translocation of FoXO3 to the nucleus and induced autophagy [47]. Inversely, many studies reported FoXO1 activation is not affected by mTOR signaling – RAPA was unable to inhibit the phosphorylation of FoXO1 [48] and FoXO1 translocation [45]. In the current study, we found that DEX stimulated MuRF1 meanwhile suppressing mTOR. Moreover, DEX-induced MuRF1 overexpression was restored when pretreated with RAPA. These results suggest that GCs exert both a catabolic and an anti-anabolic action. When further suppressing anabolic pathway, the enhanced catabolism was attenuated, demonstrating a systemic compensation mechanism.

In conclusion, the present study indicates that excessive GCs increased UPS-dependent protein proteolysis via stimulating MuRF1, but not atrogin-1. GCs induced MuRF1 overexpression independent of Akt/FoXO1 pathway.

## Abbreviations

Akt: also known as Protein Kinase B
DEX: dexamethasone
DMEM: Dulbecco’s modified eagle medium
EPOX: epoxomicin
FoxO1: forkhead box O1
GC: glucocorticoid
GR: GC receptor
GRE: GC response element
HRP: horse radish peroxidase
KLF15: Kruppe-like factor 15
LY: LY294002
mTOR: mammalian target of rapamycin
MuRF1: muscle-specific RING finger protein 1
RAPA: rapamycin
RT-PCR: reverse transcription-polymerase chain reaction
UPS: ubiquitin-proteasome system
